# Ecological determinants of mean family age of angiosperm trees in forest communities in China

**DOI:** 10.1038/srep28662

**Published:** 2016-06-29

**Authors:** Hong Qian, Shengbin Chen

**Affiliations:** 1 Research and Collections Center, Illinois State Museum, 1011 East Ash Street, Springfield, IL 62703, USA; 2 Nanjing Institute of Environmental Sciences, Ministry of Environmental Protection, 8 Jiangwangmiao Street, Nanjing, 210042, China

## Abstract

Species assemblage in a local community is determined by the interplay of evolutionary and ecological processes. The Tropical Niche Conservatism hypothesis proposes mechanisms underlying patterns of biodiversity in biological communities along environmental gradients. This hypothesis predicts that, among other things, clades in areas with warm or wet environments are, on average, older than those in areas with cold or dry environments. Focusing on angiosperm trees in forests, this study tested the age-related prediction of the Tropical Niche Conservatism hypothesis. We related the mean family age of angiosperm trees in 57 local forests from across China with 23 current and paleo-environmental variables, which included all major temperature- and precipitation-related variables. Our study shows that the mean family age of angiosperm trees in local forests was positively correlated with temperature and precipitation. This finding is consistent with the age-related prediction of the Tropical Niche Conservatism hypothesis. Approximately 85% of the variance in the mean family age of angiosperm trees was explained by temperature-related variables, and 81% of the variance in the mean family age of angiosperm trees was explained by precipitation-related variables. Climatic conditions at the Last Glacial Maximum did not explain additional variation in mean family age after accounting for current environmental conditions.

Species assemblage in a local community is determined by the interplay of evolutionary and ecological processes. Species in a local community are assembled from a regional species pool. Which species in a regional species pool are assembled into a local community depends on the dispersal ability of species in the regional species pool that allows them to reach the local community, and the degree to which the species that have reached the local community can tolerate abiotic and biotic conditions in the local community^1^. It is broadly accepted that ecological traits (e.g., cold and drought tolerance) are phylogenetically conserved^2^,^3^,^4^. Thus, species composition in local communities should reflect the imprint of phylogenetic inertia.

The Tropical Niche Conservatism hypothesis proposes mechanisms underlying patterns of biodiversity and phylogenetic structure in biological communities^5^. This hypothesis posits that ecological niches are phylogenetically conserved, most of the land surface of Earth was under tropical climates during the time when many clades (including most of extant angiosperm families) originated, and temperate environments have existed only since the global cooling initiated in the Eocene and thus is much younger than tropical environments. The hypothesis predicts that with decreasing environmental temperature and moisture, the mean clade age of species in an assemblage is younger, and the species are phylogenetically more closely related.

Few studies have tested the prediction of the Tropical Niche Conservatism hypothesis with respect to the relationship between clade age and climate for angiosperm species in local forest communities and the results of these studies are mixed. For example, Hawkins *et al*. found that in North America forest communities in lower latitudes are dominated by angiosperm trees from older families whereas those in higher latitudes are dominated by angiosperm tress from younger families^6^, a finding consistent with the prediction of the Tropical Niche Conservatism hypothesis. Similarly, Qian found that along a latitudinal gradient in South America, forest communities in warmer latitudes are dominated by angiosperm trees from older families^7^, which is consistent with the finding of Hawkins *et al*.^6^ and the prediction of the Tropical Niche Conservatism hypothesis. However, Qian also found that along an elevational gradient in tropical South America, forest communities in warmer (lower) elevations are dominated by angiosperm trees from younger families^7^; this finding is contrary to the prediction of the Tropical Niche Conservatism hypothesis. All the previous studies on the relationship between mean family age and climatic variables for angiosperm species in local forest communities were conducted for assemblages in the New World. There is a need to investigate how mean family age is related with climatic variables in other regions of the world.

Here, we conduct a test on the relationship between mean family age and climatic variables for angiosperm tree species in local forest communities in China. To our knowledge, this is the first such test for local forest communities not only for Asia but also for the Eastern Hemisphere. Unlike previous tests examining only few climatic variables, we include a large number of temperature- and precipitation-related variables in our analyses. We also investigate whether differences in temperature and precipitation between the Last Glacial Maximum (LGM) and the present influence mean clade age after accounting for current climatic conditions.

## Results

**Figure 1:**
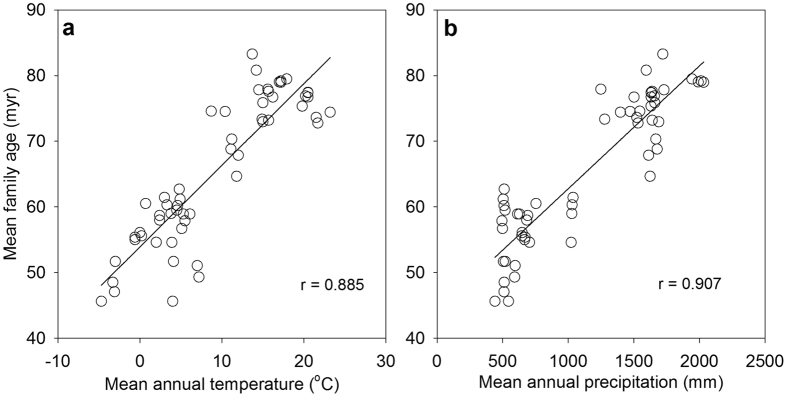
Relationship between mean family age (million years, myr) and mean annual temperature (BIO1) or mean annual precipitation (BIO12) for angiosperm tree species in forests.

Mean family age varied greatly among the forest sites, ranging from 45.63 to 83.27 million years (myr), and was strongly and negatively correlated with latitude (r = −0.896). BIO1, BIO2, BIO4, BIO6, and BIO11 were the temperature-related variables that were strongly correlated with mean family age (|r| ≥ 0.85 in all cases) while BIO12 and BIO15 were the precipitation-related variables that were strongly correlated with mean family age (|r| ≥ 0.85 in both cases; Table 1; Fig. 1). Mean family age was correlated with AET more strongly than with PET (r = 0.911 versus 0.807; Table 1). Standard deviation of family ages of species in each plot was significantly and positively correlated with mean annual temperature and annual precipitation (r = 0.518 and 0.555, respectively; p < 0.001 in both cases), and was significantly and negatively correlated with latitude (r = −0.488; p < 0.001).

The positive relationship between mean family age and temperature holds when elevation was taken into account. Specifically, when mean family age was regressed on mean annual temperature with elevation included as a covariate, regression coefficient for mean annual temperature was 0.126 (p < 0.001) and regression coefficient for elevation was not significant (p = 0.297). When only forest plots located in low elevations (<500 m) were included in an analysis, the correlation between mean family age and annual mean temperature was positive and significant (r = 0.910, p < 0.001).

**Figure 2:**
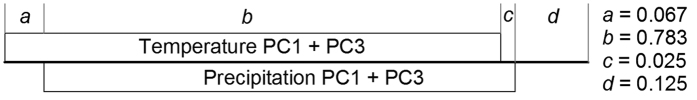
Partitioning the variance in mean family age in forest communities between temperature (PC1 and PC3) and precipitation (PC1 and PC3) variables. *a* and *c* represent the variance independently explained by temperature and precipitation variables, respectively, *b* represents the variance jointly explained by temperature and precipitation variables, and *d* represents the variance not explained by climatic variables.

The first three principal components (PC) of the 11 temperature-related variables explained 97.6% of the variation in the 11 temperature-related variables with PC1 of the temperature-related variables alone explaining 84.2% of the variation. When mean family age was regressed on these three temperature-related PCs simultaneously, PC2 was not significant (p > 0.05). PC1 and PC3 of the temperature-related variables together explained 85.0% of the variance in mean family age, based on r^2^_adj_ (Fig. 2).

The first three PCs of the precipitation-related variables explained 98.4% of the variation in the eight precipitation-related variables, and the first PC of the precipitation-related variables alone explained 87.9% of the variation. When mean family age was regressed on these three precipitation-related PCs, PC2 was not significant (p > 0.05). PC1 and PC3 of the precipitation-related variables together explained 80.8% of the variance in mean family age (Fig. 2).

When mean family age was simultaneously regressed against temperature-related PC1 and PC3 and precipitation-related PC1 and PC3, they together explained 87.5% of the variance in mean family age. The two temperature-related PCs and two precipitation-related PCs jointly explained 78.3% of the variance in mean family age whereas the two temperature-related PCs independently explained 6.7% and the two precipitation-related PCs independently explained 2.5% of the variance in mean family age (Fig. 2).

When temperature anomaly and temperature velocity were included in the model that included the PC1 and PC3 of current temperature and the PC1 and PC3 of current precipitation, the two metrics of paleoclimate did not explain additional variation in mean family age (r^2^_adj_ = 0.870).

## Discussion

We analyzed a set of 57 local forest communities to determine patterns of mean family age for angiosperm trees along latitudinal and environmental gradients. To our knowledge, there are only two published studies that have investigated the relationship between mean family age and climate (mainly temperature) for angiosperms in local forest communities along latitudinal gradients, and both studies were conducted for forest communities in the New World^6^,^7^. Furthermore, either study was more or less constrained to only one climatic zone, e.g., Hawkins *et al*.’s study covered temperate latitudes^6^ whereas Qian’s study covered tropical latitudes^7^. The present study covered both tropical and temperate latitudes. Our analysis shows striking patterns in mean family age: the angiosperm components of forest communities are dominated by trees from younger families in more northern latitudes and colder or drier environments. The fact that the degree of variation in family ages of species is significantly and negatively correlated with latitude suggests that tropical sites contain a mix of young and old families whereas temperate sites contain primarily younger families. Using the same set of forest plots as in the present study, Qian *et al*. found that phylogenetic relatedness of angiosperm tree species in local communities increases with decreasing environmental temperature and precipitation^8^. Thus, the findings of both the present and Qian *et al*.’s studies^8^ are consistent to the predictions of the Tropical Niche Conservatism hypothesis.

Previous studies on the relationship between mean family age and climate generally focused on minimum temperature (i.e., temperature in the coldest month). Correlation coefficient between mean family age and minimum temperature is 0.672 for angiosperm trees in local forest communities in North America^6^, and is 0.715 for angiosperm woody plants in local forest communities along a latitudinal gradient in South America^7^. The present study showed that the correlation coefficient between mean family age and minimum temperature (i.e., BIO6 in this study) is 0.892 (Table 1), which is much higher than those for American forest communities. It is well known that the relationship between macroecological patterns and environmental measures is generally weaker at a finer spatial scale. However, the relationship between mean family age and minimum temperature that was observed in the present study for angiosperm tree assemblages at a fine scale (0.1 ha per sampling unit) in China is much stronger than that for angiosperm tree assemblage at a broad, regional scale (12,100 km^2^ per sampling unit) in North America, for which correlation coefficient ranged from 0.28 to 0.77, depending on longitude^9^. The strong relationship between mean family age and minimum temperature in the Chinese data set is likely partly because the Chinese data set covered a longer thermal gradient than those used in American analyses and partly because distributions of plant species in eastern Asian flora are at equilibrium with current climate at a greater degree than those in North America due to much less impact of the Last Glacial Maximum in Asia^10^. Our analysis showed that paleoclimate of the Last Glacial Maximum did not explain any variation in mean family age that was not explained by the current climate.

Temperature and precipitation both are strongly correlated with mean family age in the present study. Because temperature and precipitation variables are also strongly correlated with each other (e.g., r = 0.852 between BIO1 and BIO12), the effect of temperature-related variables on mean family age largely overlaps with that of precipitation-related variables. However, temperature-related variables independently explained more variation in mean family age than did precipitation-related variables (6.7% versus 2.5%). This finding is consistent with previous studies showing that temperature plays a more important role than precipitation in shaping macroecological patterns of plants^11^ and animals^12^. In our study, the fact that temperature-related variables explained more variation in mean family age than did precipitation-related variables may also partly result from the larger variation of temperature-related variables among the study forest sites, compared to that of precipitation-related variables. For example, coefficient of variation for mean annual temperature in our data set is larger than that for mean annual precipitation by a factor of 1.82 (i.e., 86.2% versus 47.3%). The Tropical Niche Conservatism hypothesis considers both temperature and precipitation as major drivers of patterns of species richness and membership of community assemblages. A more complete understanding of relative roles of temperature and precipitation in driving community assembly requires analyses that will include community assemblages for which temperature and precipitation vary at a similar degree.

## Methods

Sixty 0.1-ha (20 × 50 m) forest plots were sampled in 15 areas (as shown in Appendix S1 of Qian *et al*.^8^), 14 of which are nature reserves. These areas spanned across a wide latitudinal gradient (35°) from tropical rain forests to boreal forests. For each forest plot, latitude, longitude and elevation were recorded. Woody individuals with diameter at breast height of 3 cm or larger were identified to species. All species included in this study are native tree species. Botanical nomenclature at the species level was standardized according to the Flora of China^13^. Three forest plots, each with fewer than two angiosperm tree species, were excluded. The remaining 57 plots contained 462 angiosperm tree species.

Family names for the species were standardized according to Davies *et al*.^14^ and family ages were obtained from the website (https://github.com/camwebb/tree-of-trees/blob/master/megatrees_other/davies2004.ages). For each species in each forest plot, we assigned it the age of the family to which the species belongs, and calculated mean family age (MFA) as the sum of family ages of all species in the forest plot divided by the number of species in the forest plot, a method which was used in previous studies^6^,^9^.

We related MFA to a large number of environmental variables. We obtained climatic data for each forest plot from the WorldClim database (http://www.worldclim.org)^15^. All the 19 biological climatic variables (i.e., BIO1 through BIO19) in the WorldClim database were used in the present study. They are annual mean temperature (BIO1), mean diurnal range (BIO2), isothermality (BIO3), temperature seasonality (BIO4), maximum temperature of the warmest month (BIO5), minimum temperature of the coldest month (BIO6), temperature annual range (BIO7), mean temperature of the wettest quarter (BIO8), mean temperature of the driest quarter (BIO9), mean temperature of the warmest quarter (BIO10), mean temperature of the coldest quarter (BIO11), annual precipitation (BIO12), precipitation of the wettest month (BIO13), precipitation of the driest month (BIO14), precipitation seasonality (BIO15), precipitation of the wettest quarter (BIO16), precipitation of the driest quarter (BIO17), precipitation of the warmest quarter (BIO18), and precipitation of the coldest quarter (BIO19). The first 11 variables (i.e., BIO1 through BIO11) are temperature-related variables whereas the last eight variables (i.e., BIO12 through BIO19) are precipitation-related variables. We conducted a principal component analysis (PCA) for the 11 temperature-related variables to generate a small number of synthetic variables (principal components, PC) to represent the temperature-related variables (Appendix S1). We conducted a second principal component analysis for the eight precipitation-related variables to generate a small number of synthetic variables to represent the precipitation-related variables (Appendix S2). Both PCA were based on correlation matrix.

In addition to relating MFA to the 19 climatic variables, we also related MFA to annual actual evapotranspiration (AET) and annual potential evapotranspiration (PET), which are two commonly used environmental variables in macroecological studies. We obtained data for AET and PET from CGIAR-CSI’s global aridity and PET database and global high-resolution soil-water balance database (http://www.cgiar-csi.org/data)^16^,^17^.

We also investigated the extent to which paleoclimatic conditions at the Last Glacial Maximum (LGM) influenced patterns of MFA after accounting for current climatic conditions. We used two metrics to quantify paleoclimatic conditions: glacial-interglacial mean annual temperature anomaly and glacial-interglacial mean annual temperature velocity. Mean annual temperature anomaly was calculated by subtracting LGM mean annual temperature from current mean annual temperature, and mean annual temperature velocity was calculated by dividing the ratio between the rate of climate change through time by the rate of climate change across space^18^. The mean value was computed for the LGM mean annual temperature from that of the Community Climate System Model version 3 (CCSM3)^15^,^19^ and Model for Interdisciplinary Research on Climate version 3.2 (MIROC3.2)^20^ palaeoclimatic simulations for the LGM.

We used correlation analysis and regression analysis to assess the relationship between MFA and environmental variables. We assessed the strength of the MFA–environment relationship based on Pearson’s correlation coefficient and adjusted coefficient of determination (r^2^_adj_). We conducted variation partitioning analysis to determine the variation in MFA that was explained independently by temperature-related variables, independently by precipitation-related variables, and jointly by temperature- and precipitation-related variables. In addition to calculating traditional p-values in significance tests, we also calculated p*-*values based on geographically effective degrees of freedom to account for spatial autocorrelation. Specifically, we used the approach of Dutilleul^21^ to determine p*-*values after accounting for spatial autocorrelation.

To account for the effect of elevation on the relationship between MFA and temperature^7^, we conducted two analyses. First, we regressed MFA on annual mean temperature and included elevation as a covariate in the regression using all 57 forest plots. Second, we regressed MFA on annual mean temperature only for the forest plots located in low elevations (<500 m). PC-ORD was used to conduct PCA^22^. All other analyses were conducted using SAM v4.0 (http://www.ecoevol.ufg.br/sam/)^23^.

## Additional Information

**How to cite this article**: Qian, H. and Chen, S. Ecological determinants of mean family age of angiosperm trees in forest communities in China. *Sci. Rep.* **6**, 28662; doi: 10.1038/srep28662 (2016).

## Supplementary Material

Supplementary Information

## Figures and Tables

**Table 1:** Correlation between mean family age and environmental variables.

Variable	R	p-value	p-value*
BIO1	0.885	<10^−9^	0.038
BIO2	−0.903	<10^−9^	0.033
BIO3	0.433	<10^−3^	0.335
BIO4	−0.863	<10^−9^	0.053
BIO5	0.715	<10^−9^	0.153
BIO6	0.892	<10^−9^	0.039
BIO7	−0.875	<10^−9^	0.049
BIO8	0.535	<10^−4^	0.168
BIO9	0.899	<10^−9^	0.035
BIO10	0.825	<10^−9^	0.049
BIO11	0.886	<10^−9^	0.040
BIO12	0.907	<10^−9^	0.022
BIO13	0.832	<10^−9^	0.057
BIO14	0.849	<10^−9^	0.045
BIO15	−0.850	<10^−9^	0.054
BIO16	0.857	<10^−9^	0.029
BIO17	0.832	<10^−9^	0.053
BIO18	0.684	<10^−8^	0.100
BIO19	0.834	<10^−9^	0.050
AET	0.911	<10^−9^	0.020
PET	0.807	<10^−9^	0.066

See Materials and Methods for abbreviations of environmental variables. p-values* were determined based on geographically effective degrees of freedom after accounting for spatial autocorrelation.
