# Supplementary material for: Ecological determinants of mean family age of angiosperm trees in forest communities in China
Source: Sci Rep. 2016 Jun 29;6:28662. doi: 10.1038/srep28662 (PMC4926104; doi:10.1038/srep28662)
Supplement: Supplementary Information [file srep28662-s1.pdf]

Supplementary information

Ecological determinants of mean family age of angiosperm trees in forest communities in China

Hong Qian & Shengbin Chen

Content

Appendix S1. Results of a principal component analysis based on the correlation matrix between temperature-related variables of each plot.

Appendix S2. Results of a principal component analysis based on the correlation matrix between precipitation-related variables of each plot.

Appendix S1. Results of a principal component analysis based on the correlation matrix between temperature-related variables of each plot (n = 57).

	PCA axis		
	PC1	PC2	PC3
Eigenvalue	9.267	0.858	0.608
Percentage of variance	0.842	0.078	0.055
Cumulative %	0.842	0.921	0.976
Eigenvectors			
BIO1	-0.328	0.004	0.022
BIO2	0.305	0.042	-0.410
BIO3	-0.219	-0.498	-0.737
BIO4	0.316	0.270	-0.112
BIO5	-0.268	0.550	-0.099
BIO6	-0.326	-0.096	0.117
BIO7	0.318	0.230	-0.157
BIO8	-0.255	0.417	-0.457
BIO9	-0.325	-0.061	0.115
BIO10	-0.308	0.355	-0.038
BIO11	-0.326	-0.101	0.071

Appendix S2. Results of a principal component analysis based on the correlation matrix between precipitation-related variables of each plot (n = 57).

	PCA axis		
	PC1	PC2	PC3
Eigenvalue	7.035	0.626	0.213
Percentage of variance	0.879	0.078	0.027
Cumulative %	0.879	0.958	0.984
Eigenvectors			
BIO12	-0.373	0.082	-0.111
BIO13	-0.343	0.479	-0.349
BIO14	-0.364	-0.304	-0.028
BIO15	0.344	0.413	-0.269
BIO16	-0.354	0.382	-0.307
BIO17	-0.364	-0.309	0.032
BIO18	-0.326	0.423	0.801
BIO19	-0.360	-0.285	-0.236
